# Bilingualism in older Mexican-American immigrants is associated with higher scores on cognitive screening

**DOI:** 10.1186/s12877-016-0368-1

**Published:** 2016-11-24

**Authors:** Claudia Padilla, Mario F. Mendez, Elvira E. Jimenez, Edmond Teng

**Affiliations:** 1 Department of Neurology, David Geffen School of Medicine, University of California, Los Angeles, CA USA; 2 Department of Psychiatry & Biobehavioral Sciences, David Geffen School of Medicine, University of California, Los Angeles, CA USA; 3 Veterans Affairs Greater Los Angeles Healthcare System, Los Angeles, CA USA

**Keywords:** Bilingualism, Cognitive reserve, Cognitive decline, Aging, SALSA

## Abstract

**Background:**

Bilingualism may protect against cognitive aging and delay the onset of dementia. However, studies comparing monolinguals and bilinguals on such metrics have produced inconsistent results complicated by confounding variables and methodological concerns.

**Methods:**

We addressed this issue by comparing cognitive performance in a more culturally homogeneous cohort of older Spanish-speaking monolingual (*n* = 289) and Spanish-English bilingual (*n* = 339) Mexican-American immigrants from the Sacramento Longitudinal Study on Aging.

**Results:**

After adjusting for demographic differences and depressive symptoms, both groups performed similarly at baseline on verbal memory but the bilingual group performed significantly better than the monolingual group on a cognitive screening test, the Modified Mini-Mental State Examination (3MS; *p* < 0.001). Group differences on the 3MS were driven by language/executive and language/praxis factors. Within the bilingual group, neither language of testing nor degree of bilingualism was significantly associated with 3MS or verbal memory scores. Amongst individuals who performed in the normal or better range on both tests at baseline and were followed for an average of 6 years, both monolinguals and bilinguals exhibited similar rates of cognitive decline on both measures.

**Conclusions:**

These findings suggest that bilingualism is associated with modest benefits in cognitive screening performance in older individuals in cross-sectional analyses that persist across longitudinal analyses. The effects of bilingualism should be considered when cognitively screening is performed in aging immigrant populations.

**Electronic supplementary material:**

The online version of this article (doi:10.1186/s12877-016-0368-1) contains supplementary material, which is available to authorized users.

## Background

Recent census data indicate that multiple languages are spoken in approximately 20% of American households. The proportion of such households has doubled over the past 30 years, with the largest increases among households in which both Spanish and English are spoken [1]. These findings emphasize the growing prevalence of bilingualism in the United States.

Of particular interest are the potential effects of bilingualism on cognitive function. Prior work suggests that bilinguals perform better than monolinguals on a subset of tests of executive function [2]. Neuroimaging studies demonstrate greater frontal lobe white matter volumes [3] and structural integrity [4, 5] as well as more robust frontal-executive processing networks [6] in bilinguals relative to monolinguals. These findings encompass a wide range of white matter tracts, particularly those implicated in executive and language functions, and may be attributable to the involvement of frontal regions and executive networks when switching between languages. However, the potential benefits of bilingualism on executive functioning may have been exaggerated by methodological issues and restricted to specific bilingual subpopulations and/or certain types of executive tasks [7, 8].

When the potential benefits of bilingualism on executive function have been studied in the context of aging, effect sizes have been larger in older cohorts than younger cohorts [9], as older bilinguals may have developed greater cognitive reserve [10]. One theory of cognitive reserve postulates that it represents the flexible use of neuronal networks to compensate for diminished brain integrity associated with aging and/or disease [11]. It may be more pronounced in bilingual individuals due to the more highly developed frontal-executive networks needed to rapidly switch between languages and thus modulate their risk for dementia relative to monolinguals [10, 12]. A number of studies report delays of up to 5 years in the onset of dementia or cognitive impairment in multilingual versus monolingual individuals [13–19]. Likewise, more Alzheimer’s disease (AD) associated brain atrophy is seen in bilinguals relative to monolingual controls, suggesting that bilinguals maintain a similar level of cognitive performance despite a greater degree of underlying AD pathology [20]. Nevertheless, other studies have failed to replicate these findings [21–26].

These conflicting findings may result in part from methodological differences between studies, such as the use of different cognitive assessments and statistical analyses, and/or potential confounding effects of environmental variables, such as immigration, education, socioeconomic status, and cultural factors [7]. Therefore, we sought to specifically investigate the influence of bilingualism on cognition in Mexican-Americans, a culturally distinct Spanish-speaking population. Although recent work has failed to show a protective effect of Spanish-English bilingualism on cognitive decline in Caribbean Hispanic immigrants [26], that cohort was more culturally heterogeneous, with participants from multiple Caribbean countries. In the current study, we examined the associations between bilingualism and both cross-sectional and longitudinal cognitive performance in elderly Mexican-American immigrants enrolled in the Sacramento Latino Study on Aging (SALSA). We sought to use this more homogeneous study cohort to determine whether bilingualism modulates cognitive function and the potential effects of language of testing and degree of bilingualism, both on a more general test of cognitive screening and on a more specific test of verbal memory.

## Methods

### Participants

We analyzed data from the SALSA database (https://www.icpsr.umich.edu/icpsrweb/icpsr/series/247), which includes participants recruited from the Sacramento metropolitan region and surrounding counties. The overall SALSA cohort represents an unstratified epidemiological sample that includes 1,789 community-dwelling participants who were ≥ 60 years of age and self-identified as Latino, who could be either cognitively intact or cognitively impaired at baseline [27]. We restricted our analyses to participants who were ≥ 65 years of age at baseline and born in Mexico (*n* = 628). Data collection was completed in either Spanish or English, per participant’s choice. The University of California Davis Institutional Review Board approved all study procedures. Informed consent was obtained from each participant.

### Language status

Participants were dichotomized as monolingual or bilingual using a language question from the Acculturation Rating Scale for Mexican Americans-II (ARSMA-II; [28]): “Do you speak English?” Spanish-speaking monolinguals were defined as those who answered “not at all.” Bilingualism was defined as and stratified by answers of “not very often,” “very often” or “almost always.”

### Measures of depressive symptoms and cognitive function

Depressive symptoms were measured with the Center for Epidemiologic Studies Depression Scale (CES-D); higher scores indicate more depressive symptomatology [29]. Global cognitive functioning was assessed with a widely used cognitive screening test, the Modified Mini-Mental State Examination (3MS; range 0–100) [30]. The 3MS has previously been validated in both Spanish and English and item response theory indicates that it performs similarly in both languages [31]. A prior factor analysis of the 3MS in a non-demented cohort yielded four factors: verbal memory/fluency, language/executive, orientation/visuoconstruction, and language/praxis [32]. Verbal memory was assessed with the Spanish English Verbal Learning Test (SEVLT), a verbal list-learning task with equivalent Spanish and English versions [33]. SEVLT performance was analyzed using total scores on learning trials 1–5 (range 0–75) and delayed recall (range 0–15). Longitudinal 3MS and SEVLT data were available for a subset of participants, for whom annualized change scores were calculated. The psychometrists administering these tests were predominantly of bicultural Mexican-American heritage [31]. SALSA also includes selected subtests from Spanish and English Neuropsychological Assessment Scales (SENAS). However, since SENAS indices were only administered to a limited subset of participants that was not representative of the larger cohort, they were excluded from our analyses.

### Statistical analyses

Statistical analyses were performed using SPSS 20.0 for Mac (IBM, Armonk NY). Demographic and CES-D data were compared across groups using independent samples *t*-tests and analysis of variance (ANOVA) for continuous variables and chi-square tests for categorical variables. Baseline and longitudinal cognitive data were compared between groups using analysis of covariance (ANCOVA) adjusted for baseline differences in demographic and depressive indices. There were scattered missing data points for baseline CES-D scores and monthly household income (see Tables 1 and 3 for details), resulting in smaller samples sizes for adjusted versus unadjusted comparisons.

**Table 1:** Baseline demographic data for monolingual and bilingual groups

	Monolingual	Bilingual	*t*/*χ* ^2^
N	289	339	
Age	73.6 (6.9)	74.3 (6.7)	−1.33
Gender (% male)	35.9%	48.6%	10.25*
Years of Education	2.2 (2.7)	6.3 (4.6)	−13.54*
CES-D score^a^	13.6 (11.2)	9.7 (10.3)	4.40*
Monthly Household Income^b^			*χ* ^2^(4,610) = 82.24*
<$1000	78.0%	43.5%	
$1000–$1499	14.8%	30.3%	
$1500–$1999	5.4%	11.7%	
$2000–$2499	1.8%	6.6%	
≥$2500	0.0%	7.8%	

Parentheses represent standard deviation. *p < 0.05. ^a^Baseline CES-D data was missing for 15 monolingual and 17 bilingual participants. ^b^Baseline monthly household income data was missing for 12 monolingual and 6 bilingual participants

## Results

### Demographics

Baseline demographic data for the monolingual (*n* = 289) and bilingual (*n* = 339) groups are shown in Table 1. The groups were similar in age (*p* = 0.19), but the bilingual group had more men (*p* = 0.001), more years of formal education (*p* < 0.001) and higher monthly household incomes (*p* < 0.001). Baseline CES-D scores (Table 1) showed significantly more depressive symptoms in monolinguals relative to bilinguals (*p* < 0.001). Using the standard CES-D cutoff score of ≥16 [29], higher rates of clinically significant depression [*χ* ^2^(1,596) = 13.83, *p* < 0.001] were seen in monolinguals (38.3%) versus bilinguals (24.2%). These results confirm prior Latino immigrant studies reporting increased depressive symptoms in individuals with lower levels of English language use and acculturation [34, 35]. Analyses of self-reported past medical history in the two groups revealed similar prevalence for diabetes [monolingual: 28.4%, bilingual: 24.2%; *χ* ^2^(1,628) = 1.65, *p* = 0.20] and hypertension [monolingual: 47.9%, bilingual: 43.6%; *χ* ^2^(1,623) = 1.14, *p* = 0.29]. However, significantly higher rates of prior stroke [*χ* ^2^(1,628) = 5.84, *p* = 0.016] were reported by bilinguals (10.9%) relative to monolinguals (5.5%), which may reflect poorer knowledge of stroke symptoms among Spanish-speaking versus English-speaking Hispanics [36].

Within the bilingual group, English was spoken “not very often” by 60% (*n* = 202), “very often” by 13% (*n =* 44), and “almost always” by 27% (*n* = 93) of participants. When the bilingual group was stratified by frequency of English use, similar proportions of men were seen in each subgroup [“not very often”: 48.5%; “very often”: 59.1%; “almost always”: 44.1%; *χ* ^2^(2,339) = 2.70, *p* = 0.26]. 79% (*n* = 269) of these individuals underwent testing in Spanish, particularly those who reported less frequent English usage [“not very often”: 96.5%; “very often”: 81.8%; “almost always”: 40.9%; *χ* ^2^(2,339) = 120.66, *p* < 0.001]. Baseline demographic information for these bilingual subgroups are shown in Additional file 1: Table S1 and Additional file 2: Table S2. Age [*F*(2,336) = 1.77, *p* = 0.17] and gender distribution [*χ* ^2^(2,339) = 2.70, *p* = 0.26] did not differ across different levels of bilingual proficiency. However, higher levels of education [*F*(2,336) = 54.03, *p* < 0.001] and monthly household incomes [*χ* ^2^(8,333) = 39.81, *p* < 0.001] coincided with higher frequency of spoken English. Likewise, while bilingual participants tested in English were similar in gender distribution to those tested in Spanish [*χ* ^2^(1,339) = 0.08, *p* = 0.77], they were significantly older [*t*(337) = 2.95, *p* = 0.003] and had higher levels of education [*t*(337) = 5.73, *p* < 0.001] and monthly household incomes [*χ* ^2^(4,333) = 28.59, *p* < 0.001]. Given these demographic differences, subsequent comparisons between monolingual and bilingual groups were performed with and without adjustment for baseline age, gender, education, income, and CES-D scores.

### Baseline 3MS

**Fig. 1:**
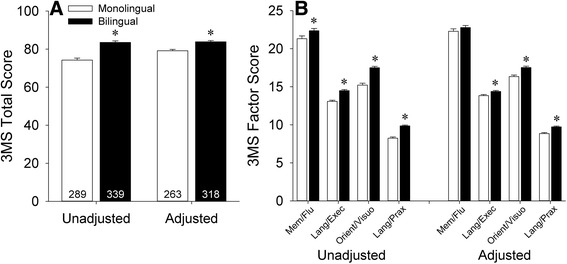
Baseline unadjusted and adjusted (**a**) total and (**b**) factor scores on the Modified Mini-Mental State Examination (3MS) in monolinguals versus bilinguals. Error bars represent standard error of the mean (SEM). *p < 0.05 versus the monolingual group. Mem/Flu: verbal memory/fluency factor; Lang/Exec: language/executive factor; Orient/Visuo: orientation/visuoconstruction factor; Lang/Prax: language/praxis factor

Baseline total 3MS scores for the monolingual and bilingual groups are shown in Fig. 1a. Significantly better 3MS performance was seen in the bilingual group relative to the monolingual group both before [*t*(626) = −7.25, *p* < 0.001] and after adjustment for demographic variables and baseline CES-D scores [*F*(1,573) = 20.76, *p* < 0.001]. 3MS performance was not affected by language of administration, as similar scores were seen in bilinguals tested in Spanish versus English [unadjusted: *t*(337) = −0.50, *p* = 0.62; adjusted: *F*(1,310) = 1.56, *p* = 0.21]. Likewise, in analyses stratified by self-reported English usage, similar performances on the 3MS were seen amongst bilinguals who spoke English “almost always,” “very often,” or “not very often” in both unadjusted [*F*(2,336) = 2.68, *p* = 0.07] and adjusted [*F*(2,308) = 1.33, *p* = 0.27] analyses.

We subsequently examined performance of the monolingual and bilingual groups on previously identified 3MS factors (Fig. 1b) [32]. The bilingual group performed better in both raw and adjusted analyses of the language/executive [unadjusted: *t*(626) = −6.86, *p* < 0.001; adjusted: *F*(1,573) = 7.26, *p* = 0.007], orientation/visuospatial [unadjusted: *t*(626) = −7.53, *p* < 0.001; adjusted: *F*(1,573) = 18.35, *p* < 0.001], and language/praxis [unadjusted: *t*(626) = −8.61, *p* < 0.001; adjusted: *F*(1,573) = 22.34, *p* < 0.001] factors. However, on the verbal memory/fluency factor, the bilingual advantage seen on unadjusted analyses [*t*(626) = −2.26, *p* = 0.024] failed to survive adjustment for demographic variables and CES-D scores [*F*(1,573) = 1.19, *p* = 0.28]. Logistic regression analysis (Table 2) indicated that performance on the language/executive and language/praxis factors distinguished between the monolingual and bilingual groups (*p*’s < 0.05).

**Table 2:** Logistic regression analysis of demographic variables and 3MS factors associated with bilingualism at baseline

	B	SE	Wald *χ* ^2^	OR (95% CI)	*p*
Age	0.04	0.02	5.00	1.04 (1.01–1.07)	0.025
Education	0.22	0.04	34.80	1.25 (1.16–1.34)	<0.001
Household income	0.59	0.13	19.90	1.80 (1.39–2.33)	<0.001
CES-D score	−0.01	0.01	1.27	0.99 (0.97–1.01)	0.259
Gender	−0.50	0.22	5.17	0.61 (0.40–0.93)	0.023
3MS factors					
Memory/Fluency	−0.001	0.02	0.001	1.00 (0.95–1.05)	0.976
Language/Executive	0.12	0.05	5.26	1.12 (1.02–1.24)	0.022
Orientation/Visuospatial	0.07	0.04	2.64	1.08 (0.99–1.17)	0.104
Language/Praxis	0.13	0.07	4.04	1.14 (1.00–1.30)	0.045

Nagelkerke *R* ^2^ = 0.425; *SE* standard error, *OR* odds ratio, *CI* confidence interval

### Baseline SEVLT

**Fig. 2:**
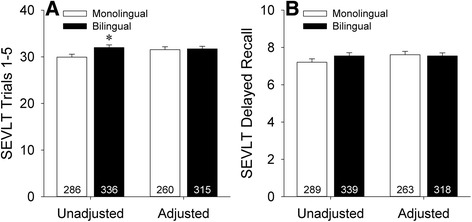
Baseline unadjusted and adjusted scores on the Spanish English Verbal Learning Test (SEVLT) for (**a**) learning trials 1–5 and (**b**) delayed recall in the monolingual and bilingual groups. Error bars represent SEM. *p < 0.05 versus the monolingual group. Learning trial data were not available for 3 participants in each group

While the 3MS measures global cognitive function, the SEVLT specifically assesses verbal learning and memory [33]. Baseline scores across SEVLT learning trials 1–5 are shown in Fig. 2a. Significantly better performance across learning trials was seen in bilinguals relative to monolinguals in unadjusted analyses [*t*(620) = −2.38, *p* = 0.017]. However, this difference did not survive adjustment for demographics and CES-D scores [*F*(1,567) = 0.04, *p* = 0.84]. Amongst bilinguals, language of testing did not affect performance in either unadjusted [*t*(334) = −1.17, *p* = 0.24] or adjusted [*F*(1,307) = 3.36, *p* = 0.07] analyses. Similarly, when SEVLT learning trial data were stratified by frequency of English usage, no effects were seen in either unadjusted [*F*(2,333) = 0.95, *p* = 0.39] or adjusted [*F*(2,305) = 2.06, *p* = 0.13] analyses. Baseline scores on SEVLT delayed recall are shown in Fig. 2b. There were no differences between groups on delayed recall either before [*t*(626) = −1.38, *p* = 0.17] or after [*F*(1,573) = 0.07, *p* = 0.80] adjustment for demographic variables and CES-D scores. In stratified analyses, delayed recall in bilinguals was not affected by language of administration [unadjusted: *t*(337) = −0.29, *p* = 0.77; adjusted: *F*(1,310) = 0.24, *p* = 0.63] or frequency of English usage [unadjusted: *F*(2,336) = 2.30, *p* = 0.10; adjusted: *F*(2,308) = 2.48, *p* = 0.09].

### Longitudinal 3MS and SEVLT

A subset of participants underwent longitudinal assessments with the 3MS and SEVLT at subsequent study visits. More bilinguals (*n* = 286; 84%) than monolinguals (*n* = 213; 74%) had longitudinal data available [*χ* ^2^(628) = 10.87, *p* = 0.001]. We analyzed annualized change scores for individuals whose baseline age- and education-adjusted performance was above the 20th percentile on both tests (i.e. in or above the normal range). Amongst participants with longitudinal data, a higher proportion of bilinguals (79%) than monolinguals (70%) met these performance criteria [*χ* ^2^(499) = 4.45, *p* = 0.035]. Demographic data for these participants are shown in Table 3. Although the two groups were similar in age (*p* = 0.53), the bilingual group had a significantly greater proportion of men (*p* = 0.027), higher levels of formal education, 3MS scores, and household income (all *p*’s < 0.001), and lower CES-D scores (*p* = 0.009). Baseline scores on the SEVLT indices and average follow-up intervals on both tests were similar between groups (all *p*’s > 0.10). Within the subgroups of monolingual and bilingual participants who performed above the 20th percentile on the 3MS and SEVLT at baseline, there were no significant differences in demographic, CES-D, or cognitive indices between those with and without subsequent longitudinal follow-up (data not shown).

**Table 3:** Baseline demographic and cognitive data for monolingual and bilingual participants underwent longitudinal assessment and whose adjusted 3MS and SEVLT score were above the 20^th^ percentile at baseline

	Monolingual	Bilingual	*t*(373)/*χ* ^2^(375)
N	150	225	
Age	73.0 (6.2)	73.4 (6.1)	−0.63
Gender (% male)	37.3%	48.9%	4.87*
Years of education	2.7 (2.9)	6.3 (4.5)	−8.47*
3MS (unadjusted)	83.8 (6.8)	88.7 (6.2)	−7.12*
3MS follow-up in years	5.6 (2.6)	5.8 (2.6)	−0.95
SEVLT Trials 1–5	34.6 (8.6)	34.9 (9.3)	−0.37
SEVLT Delayed Recall (unadjusted)	8.7 (2.3)	8.7 (2.4)	0.16
SEVLT follow-up in years^a^	5.4 (2.6)	5.7 (2.5)	−1.44
Baseline CES-D	11.7 (10.0)	8.9 (8.8)	2.62*
Baseline Monthly Household Income^b^			*χ* ^2^(4,366) = 35.76*
<$1000	70.1%	41.0%	
$1000–$1499	20.1%	30.2%	
$1500–$1999	6.9%	13.5%	
$2000–$2499	2.8%	7.2%	
≥$2500	0.0%	8.1%	

Parentheses represent standard deviation; *p < 0.05; ^a^Longitudinal SEVLT data was missing for 3 participants in the Bilingual group; ^b^Baseline monthly household income data was missing for 6 participants in the Monolingual group and 3 participants in the Bilingual group

**Fig. 3:**
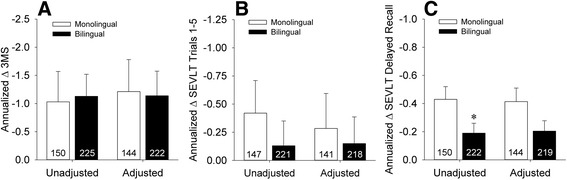
Unadjusted and adjusted annualized change scores on (**a**) total 3MS, (**b**) SEVLT learning trials 1–5, and (**c**) SEVLT delayed recall. Errors bars represent SEM. *p < 0.05 versus the monolingual group

Annualized 3MS change scores are shown in Fig. 3a. There were no differences in rates of longitudinal decline on the 3MS between the monolingual and bilingual groups either before [*t*(373) = 0.15, *p* = 0.88] or after adjustment for demographic factors and depressive symptoms [*F*(1,358) = 0.01, *p* = 0.92]. Amongst bilinguals, stratified analyses indicated that neither language of administration [unadjusted: *t*(223) = −0.43, *p* = 0.67; adjusted: *F*(1,214) = 0.37, *p* = 0.54] nor frequency of English use [unadjusted: *F*(2,222) = 0.25, *p* = 0.78; adjusted: *F*(2,212) = 0.34, *p* = 0.72] were associated with rates of longitudinal decline on the 3MS.

Annualized change scores across SEVLT learning trials are shown in Fig. 3b. There was no difference in rates of longitudinal decline on SEVLT learning trials between the monolingual and bilingual groups either before [*t*(366) = −0.81, *p* = 0.42] or after adjustment for demographic factors and depressive symptoms [*F*(1,351) = 0.11, *p* = 0.74]. Annualized change scores on SEVLT delayed recall are shown in Fig. 3c. Unadjusted analyses suggested greater declines on SEVLT delayed recall in monolinguals relative to bilinguals [*t*(370) = −2.20, *p* = 0.029]. However, this effect was attenuated by adjustment for demographic and depressive variables [*F*(1,355) = 2.73, *p* = 0.099]. Within the bilingual cohort, stratified analyses of longitudinal changes on SEVLT indices (both unadjusted and adjusted) failed to demonstrate significant effects of language of administration or frequency of English usage (all *p*’s >0.10).

## Discussion

Our analyses of first generation Mexican-American immigrants enrolled in SALSA indicate that bilinguals performed significantly better than monolinguals at baseline on global cognitive screening as measured by the 3MS, but not on immediate or delayed verbal recall memory as measured by the SEVLT. Although the groups differed on multiple demographic variables, including gender distribution, years of education, household income, and depressive symptomatology, this finding persisted after statistical adjustments for these factors. However, amongst participants who performed in or above the normal range at baseline, subsequent follow-up over approximately 6 years showed similar rates of longitudinal decline on the 3MS and SEVLT in both groups. These results suggest that bilingualism may modulate cognitive screening performance in older Mexican-American immigrants, particularly those with lower levels of formal education and/or poorer socioeconomic status.

Further analyses of the separate factors that comprise the 3MS revealed that the better performance of the bilingual group on this test was driven by better performance on the items that assess language, executive function, and praxis. These results are in concordance with neuroimaging studies that indicate that older bilinguals have greater structural integrity and functional connectivity than monolinguals in the processing networks that subserve these functions [4–6].

Our findings are also consistent with prior analyses of the more heterogeneous Caribbean Hispanic cohort of the Washington Heights-Inwood Columbia Aging Project (WHICAP). While that study included immigrants from countries with different inherent rates of Spanish-English bilingualism, it also reported that bilinguals exhibited better baseline cognitive performance than monolinguals and had similar rates of subsequent cognitive decline [26]. The WHICAP investigators postulated that while bilingualism may protect against age-associated cognitive decline, it was more likely that better baseline executive functioning facilitated bilingualism. However, bilingualism is associated with better cognitive performance in older individuals even after accounting for performance on childhood intelligence testing [17], which would seem to provide more support for the former hypothesis. Therefore, an alternative explanation for both results from our analyses and the WHICAP analyses is that the protective effects of bilingualism on general cognition may have already manifested by the time of baseline assessments (i.e. when participants have reached their mid-70’s). Future studies that incorporate more sensitive tests of executive functioning may be needed to identify subtler benefits of bilingualism on the rate of longitudinal cognitive decline.

Given that all of the bilingual participants were born in Mexico, it is perhaps unsurprising that approximately 80% of them chose to be assessed in Spanish, with the remainder choosing to be assessed in English. There were no consistent differences in cross-sectional or longitudinal performance on the 3MS or SEVLT attributable to language of testing. These results indicate that differences in cognitive performance between the monolingual and bilingual groups are unlikely to be fully explained by differences in language of testing. However, our findings diverge from a prior analysis of the larger SALSA cohort, which reported that participants tested in English exhibited poorer baseline performance than those tested in Spanish [37]. Our failure to replicate this finding may be related to our more restricted study cohort and/or different statistical approaches.

Another recent analysis of the larger SALSA cohort suggests that bilingualism provides limited, if any, protective effects in delaying dementia onset [23]. However, there is a key methodological difference between that analysis and the one reported here. Lawton and colleagues classified individuals that spoke English “not at all” and “not very often” as monolinguals [23], whereas our analyses and earlier analyses that used the same ARMSA-II item to assess language use [26] included the latter subset as bilinguals. Given that our analyses indicate that participants who reported speaking English “not very often” performed more similarly on cognitive and depressive assessments to the other subsets of the bilingual group (i.e. those that reported speaking English “very often” or “almost always”) than to the monolingual group, it remains possible that the monolingual versus bilingual classification used by Lawton and colleagues may have attenuated any differences in mean age of dementia diagnosis between groups in the larger SALSA cohort. Moreover, our results suggest that the cognitive benefits of bilingualism versus monolingualism may be relatively independent of the reported frequency of usage of the second language after controlling for other demographic factors. This is in contrast to earlier work from Gollan and colleagues that indicated lower degrees of Spanish/English bilingualism (as determined by an objective measure) were associated with earlier age of dementia onset in participants with fewer years of formal education [38]. Our failure to detect a relationship between degree of bilingualism and cognitive performance may be related to the greater imprecision of subjective measures of bilingualism, since on average, our monolingual and bilingual cohorts had levels of formal education similar to those included in Gollan and colleagues’ low education group.

Given the many challenges inherent to the study of multilingualism and cognition [7], there are a number of factors that may limit the interpretation of our findings. Although we sought to study a more homogeneous population of Latino immigrants by focusing on a Mexican-American cohort, we identified different regional patterns of immigration between the monolingual group, which is comprised of more immigrants from Western Mexico, and the bilingual group, which is comprised of more immigrants from Northern Mexico (Additional file 3: Table S3; *p* < 0.001). However, our analyses were not sufficiently powered to account for this variable. Therefore, it remains possible that we failed to account for more subtle cultural differences between the monolingual and bilingual participants arising from their native regions of origin in Mexico. In addition, SALSA does not include data regarding the age at which participants immigrated to the United States or the age at which bilinguals began speaking English. Both variables may potentially modulate the effects of multilingualism on cognition [17, 26]. Finally, there are marked differences in several demographic variables between the monolingual and bilingual participants in this subset of the SALSA database. Although we have attempted to statistically adjust for these variables, it remains possible that we may be unable to fully account for the cognitive effects of such differences due to residual confounding [7] and that such adjustments using ANCOVA may not be completely statistically valid [39]. Unfortunately, the SALSA database does not incorporate estimates of premorbid IQ or length of residence in the United States that might allow for more robust adjustments for other potential confounders for differences in cognitive performance between monolingual and bilingual participants. Nevertheless, given the difficulty in precisely matching for demographic variables in epidemiological studies of monolingualism versus bilingualism, our results may still have broader implications for the effects of the acquisition and/or usage of multiple languages in immigrant populations.

## Conclusions

Our findings add to the growing literature examining the effects of bilingualism on cognitive aging and dementia among Spanish-speaking individuals in the United States [23, 26, 38]. By limiting our focus to first-generation Mexican-American immigrants, we attempted to address a subset of potential confounds such as immigrant status [40] and cultural heterogeneity [34] that may modulate the relationship between bilingualism and age-associated cognitive decline. After additional statistical adjustments for differences in demographics and depressive symptoms, we continued to find better baseline performance among bilingual participants on the 3MS, particularly on items that assess language, praxis, and executive function. Our work complements previous studies of bilingualism that demonstrated relative benefits on some aspects of executive performance [2]. Adjustments for CES-D scores are particularly important in older Mexican-American cohorts, since prior work indicates that depressive symptoms in this demographic group are associated with poorer cross-sectional and longitudinal cognitive performance to a greater extent than in non-Latino cohorts [41–43]. The better baseline performance of bilinguals on the 3MS is consistent with prior findings of delayed onset of significant cognitive impairment in multilinguals [13–19]. Bilingual participants may be able to sustain a greater degree of cognitive deterioration before reaching a threshold that triggers a diagnosis of mild cognitive impairment or dementia, and the interpretation of cognitive screening results in such populations should take this factor into consideration.

## Additional files

Additional file 1: Table S1. Baseline demographic data for bilingual participants stratified by self-reported frequencies of use of verbal English. (DOCX 61 kb)
Additional file 2: Table S2. Baseline demographic data for bilingual participants assessed in Spanish versus English. (DOCX 52 kb)
Additional file 3: Table S3. Areas of origin for Mexican-American immigrants in the monolingual and bilingual groups. (DOCX 39 kb)

## Abbreviations

3MS: Modified mini-mental state examination
AD: Alzheimer’s disease
ANCOVA: Analysis of covariance
ANOVA: Analysis of variance
ARSMA-II: Acculturation rating scale for Mexican Americans-II
CES-D: Center for epidemiologic studies depression scale
SALSA: Sacramento Latino Study on Aging
SENAS: Spanish and English Neuropsychological Assessment Scales
SEVLT: Spanish English Verbal Learning Test
WHICAP: Washington Heights-Inwood Columbia Aging Project
