# Supplementary material for: Bilingualism in older Mexican-American immigrants is associated with higher scores on cognitive screening
Source: BMC Geriatr. 2016 Nov 24;16:189. doi: 10.1186/s12877-016-0368-1 (PMC5122008; doi:10.1186/s12877-016-0368-1)
Supplement: Additional file 1: Table S1. — Baseline demographic data for bilingual participants stratified by self-reported frequencies of use of verbal English. (DOCX 61 kb) [file 12877_2016_368_MOESM1_ESM.docx]

Supplemental Table 1. Baseline demographic data for bilingual participants stratified by self-reported frequencies of use of verbal English.

|  | Verbal English Usage | | |  |
| --- | --- | --- | --- | --- |
|  | Not Very Often | Very Often | Almost Always | *t*(337)/χ^2^(339) |
| N | 202 | 44 | 93 |  |
| Age | 73.9 (6.9) | 73.9 (6.4) | 75.4 (6.3) | 1.77 |
| Gender (% male) | 48.5% | 59.1% | 44.1% | 2.70 |
| Years of Education | 4.5 (3.7) | 7.4 (4.5) | 9.6 (4.3) | 54.03* |
| Monthly Income^a^ |  |  |  | χ^2^(8,333)=39.81* |
| <$1000 | 52.2% | 33.3% | 28.9% |  |
| $1000-$1499 | 31.3% | 33.3% | 26.7% |  |
| $1500-$1999 | 9.5% | 9.5% | 17.8% |  |
| $2000-$2499 | 3.5% | 16.7% | 8.9% |  |
| ≥$2500 | 3.5% | 7.1% | 17.8% |  |

Parentheses represent standard deviation. *p<0.05.  **^a^**Baseline monthly household income data was missing for 1 participant on the “Not Very Often” group, 2 participants in the “Very Often” group, and 3 participants in the “Almost Always” group.
