# Supplementary material for: Bilingualism in older Mexican-American immigrants is associated with higher scores on cognitive screening
Source: BMC Geriatr. 2016 Nov 24;16:189. doi: 10.1186/s12877-016-0368-1 (PMC5122008; doi:10.1186/s12877-016-0368-1)
Supplement: Additional file 2: Table S2. — Baseline demographic data for bilingual participants assessed in Spanish versus English. (DOCX 52 kb) [file 12877_2016_368_MOESM2_ESM.docx]

Supplemental Table 2. Baseline demographic data for bilingual participants assessed in Spanish versus English.

|  | Cognitive Testing Language | |  |  |
| --- | --- | --- | --- | --- |
|  | Spanish | English |  | *t*(337)/χ^2^(339) |
| N | 269 | 70 |  |  |
| Age | 73.7 (6.7) | 76.4 (6.5) |  | 2.95* |
| Gender (% male) | 49.1% | 47.1% |  | 0.08 |
| Years of Education | 5.6 (4.4) | 9.0 (4.3) |  | 5.73* |
| Monthly Income^a^ |  |  |  | χ^2^(4,333)=28.59* |
| <$1000 | 48.7% | 24.3% |  |  |
| $1000-$1499 | 30.8% | 28.6% |  |  |
| $1500-$1999 | 9.9% | 18.6% |  |  |
| $2000-$2499 | 6.1% | 8.6% |  |  |
| ≥$2500 | 4.6% | 20.0% |  |  |

Parentheses represent standard deviation. *p<0.05. **^a^**Baseline monthly household income data was missing for 6 bilingual participants tested in Spanish.
