# Supplementary material for: Bilingualism in older Mexican-American immigrants is associated with higher scores on cognitive screening
Source: BMC Geriatr. 2016 Nov 24;16:189. doi: 10.1186/s12877-016-0368-1 (PMC5122008; doi:10.1186/s12877-016-0368-1)
Supplement: Additional file 3: Table S3. — Areas of origin for Mexican-American immigrants in the monolingual and bilingual groups. (DOCX 39 kb) [file 12877_2016_368_MOESM3_ESM.docx]

Supplemental Table 3: Areas of origin for Mexican-American immigrants in the monolingual and bilingual groups.

|  | Monolingual | Bilingual |  |
| --- | --- | --- | --- |
| N | 289 | 338 |  |
| Mexican Region of Origin |  |  | χ^2^(4,627)=28.84* |
| Northern | 18.0% | 37.0% |  |
| Central | 30.1% | 24.9% |  |
| Western | 47.4% | 33.7% |  |
| Eastern | 1.7% | 1.5% |  |
| Southern | 2.8% | 3.0% |  |

**p*<0.05.
